# Interventionelle Varizentherapie mittels endoluminaler Laserablation (1940 nm) bei Patienten mit Ehlers‐Danlos‐Syndromen: zwei Fallberichte

**DOI:** 10.1111/ddg.15812_g

**Published:** 2025-11-14

**Authors:** Dennis Braß, Iliana Tantcheva‐Poór, Markus Stücker

**Affiliations:** ^1^ Klinik für Dermatologie Venerologie und Allergologie Venenzentrum der dermatologischen und gefäßchirurgischen Kliniken Katholisches Klinikum Bochum gGmbH, Bochum; ^2^ Klinik für Dermatologie und Venerologie Universität zu Köln

**Keywords:** 1940nm, Chronisch venöse Insuffizienz, Endoluminale Laserablation, Ehlers‐Danlos‐Syndrome, Krampfadern, Lasertherapie, chronic venous insufficiency, endoluminal ablation, Ehlers‐Danlos syndrome, Varicose vein

## Abstract

Die chronische venöse Insuffizienz bei Patienten mit Ehlers‐Danlos‐Syndromen stellt Ärzte vor die mitunter schwierige Indikationsstellung operativer Sanierungsmaßnahmen. Durch Gefäßfragilität kommt es insbesondere bei offenen operativen Eingriffen gehäuft zu Komplikationen wie Gefäßeinrissen oder Rupturen mit komplizierter Blutstillung und den daraus resultierenden teils lebensgefährlichen Folgen. Die Verletzlichkeit von Gefäßen unterscheidet sich in ihrer Ausprägung zwischen den verschiedenen Subtypen der Ehlers‐Danlos‐Syndrome, kann jedoch subtypübergreifend beobachtet werden. Wir führten therapeutisch die endoluminale crossennahe Laserablation insuffizienter Stammvenen bei zwei Patienten mit Ehlers‐Danlos‐Syndromen unter Verwendung einer 1940 nm Radialfaser durch und konnten hierdurch einen adäquaten Verschluss der behandelten Gefäße ohne Auftreten intra‐ oder postoperativer Komplikationen erzielen.

Das hier erstmals beschriebene Verfahren stellt eine Schnittstelle zwischen konservativer Varizentherapie und offener chirurgischer Versorgung dar und kann bei Patienten mit Ehlers‐Danlos‐Syndrom, insbesondere bei unzureichender Symptomkontrolle oder kompliziertem Krankheitsverlauf, erwogen werden.

## EINLEITUNG

Die Ehlers‐Danlos‐Syndrome sind eine heterogene Gruppe hereditärer Bindegewebserkrankungen, die derzeit in 13 Subtypen unterteilt werden.^1^ Sie zählen zu den seltenen Erkrankungen; die Inzidenz des häufigsten monogenetischen Subtyps – des klassischen Ehlers‐Danlos‐Syndroms (cEDS) – wird auf 1:20 000 geschätzt.^2^ Mutationen, welche zum Krankheitsbild führen, wurden auf 20 verschiedenen Genen gefunden und können sowohl autosomal‐dominanten als auch autosomal‐rezessiven Erbgängen folgen. Je nach Mutation verursachen die genetischen Alterationen vor allem Defekte in der Primärstruktur der fibrillären Kollagene I, III und V. Zusätzlich kann es zu Fehlfunktionen kollagenprozessierender Enzyme oder kollagenassoziierter extrazellulärer Matrix (ECM)‐Proteine wie Tenascin X, Proteoglykanen sowie beteiligter Transkriptionsfaktoren kommen.^1^, ^2^

Allen Ehlers‐Danlos‐Syndromen sind Hypermobilität der Gelenke, Hyperelastizität der Haut und Gewebsfragilität unterschiedlicher Organe (Haut, Gefäße, Hohlorgane, Auge) gemein, wobei die Ausprägung sich inner‐ und unterhalb der Subtypen unterscheidet.^2^ Trotz derzeit fehlender epidemiologischer Daten ^3^ wird über eine erhöhte Prävalenz der chronischen venösen Insuffizienz bei Ehlers‐Danlos‐Syndrom berichtet.^4^ Hiervon sind vor allem Patienten mit vaskulärem Ehlers‐Danlos‐Syndrom (vEDS) betroffen,^3^, ^5^ wobei venöse Komplikationen auch bei Patienten mit cEDS, spondylodysplastischem (spEDS) und kyphoskoliotischem Ehlers‐Danlos‐Syndrom (kEDS) beschrieben wurden.^4^ Dies stellt Phlebologen – auch angesichts des erhöhten perioperativen Risikos – vor die Schwierigkeit der Indikationsstellung operativer Behandlungsverfahren. Aktuell fehlen diesbezüglich national und international spezifische Empfehlungen.^2^ Neben den klassischen offenen operativen Verfahren stellt die endoluminale Laserablation eine minimalinvasive Möglichkeit der Stammvarizentherapie dar und wird im Folgenden an zwei Patientenkasuistiken besprochen.

## PATIENTENFALL 1

Ein 42‐jähriger Mann mit *SLC39A13*‐assoziiertem spEDS stellte sich erstmals im Jahr 2023 wegen einer chronischen venösen Insuffizienz im Stadium C4a in unserer ambulanten Sprechstunde vor. Der Patient berichtete, erstmals 1996 aufgrund von Insuffizienzen beider Venae (Vv.) saphenae magnae im Bereich der saphenofemoralen Crossen beidseits inguinal mittels Crossektomie operiert worden zu sein. Im Jahr 2009 seien aufgrund von Crossenrezidiven inguinal beidseits operative Re‐Crossektomien erfolgt. Zu diesen Eingriffen wurden keine Komplikationen beschrieben. Eine erneute Seitenastexhairese sei im Jahr 2018 begonnen worden, musste jedoch aufgrund ausgeprägter Brüchigkeit der Venen und starker Blutungen vorzeitig abgebrochen werden. Die Vorstellung in unserer Ambulanz erfolgte daraufhin mit der Frage nach möglichen Therapieoptionen bei großlumiger Seitenastvarikosis beider Ober‐ und Unterschenkel, sowie trophischen Hautveränderungen im Sinne einer *Purpura jaune d'ocre* der distalen Unterschenkel. Diagnostisch erfolgte eine duplex‐ und dopplerultraschallgestützte Untersuchung beider Beine. Hierbei zeigte sich eine Stammveneninsuffizienz der Vena (V.) saphena parva rechts (Stadium II nach Hach, Refluxdauer 2,5 s, maximaler Durchmesser 3 cm distal der Crosse: 9,9 mm), eine inkomplette Stammveneninsuffizienz der Vena saphena parva links mit kurzstreckiger aneurysmatischer Aufweitung (Refluxdauer > 2 s, maximaler Durchmesser 9,5 mm) sowie eine großlumige Seitenastvarikosis beider Ober‐ und Unterschenkel. Erneute inguinale Crossenredizive ließen sich nicht nachweisen. Aufgrund der operativen Vorgeschichte des Patienten mit einer Bindegewebsfragilität im Rahmen des vorbekannten spondylodysplastischem Ehlers‐Danlos‐Syndrom sowie der medizinischen Indikation einer Sanierung der Stammvenen‐ und Seitenastvarikosis, besprachen wir ausführlich die ambulante, minimalinvasiv‐operative, in Tumeszenzanästhesie durchgeführte, endoluminale Laserablation der rechten Vena saphena parva mittels eines 1940 nm‐Radial‐Lasers unter Verzicht auf eine Miniphlebektomie mit anschließender Sklerosierungstherapie der bestehenden Seitenastvarikosis. Intraoperativ erfolgte mit einer Gesamtenergie von 1107,5 Joule (entsprechend einer linearen Energiedosis von 185 J/cm), unter regelmäßiger Ultraschallkontrolle hinsichtlich Gefäßläsionen der langsame, konstante Rückzug der Radialfaser mittels eines Rückzugautomaten über insgesamt 149,5 s vom saphenopoplitealen Übergang nach distal. Insgesamt erfolgte so die Laserablation eines 6 cm langen insuffizienten Abschnitts der rechten V. saphena parva. Es zeigten sich keine Komplikationen im Sinne einer Gefäßbrüchigkeit oder postoperative Blutungen aus der Punktionsstelle an der rechten Wade.

**ABBILDUNG 1:**
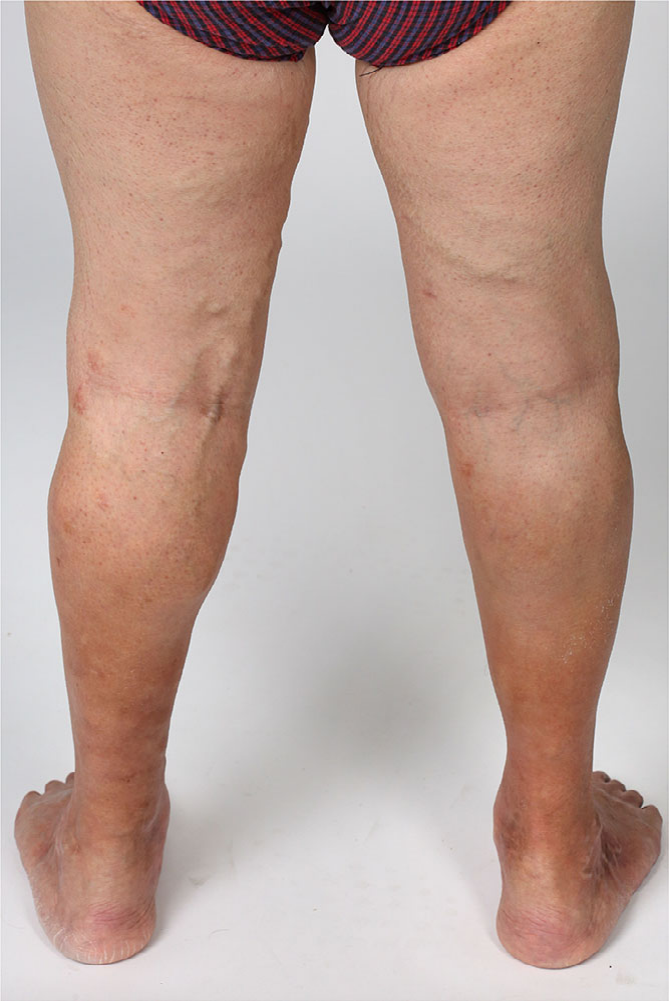
Befund bei Erstvorstellung: Ausgeprägte Seitenastvarikosis der dorsomedialen Ober‐ und Unterschenkel beidseits mit kräftiger *Purpura jaune d'ocre*.

**ABBILDUNG 2:**
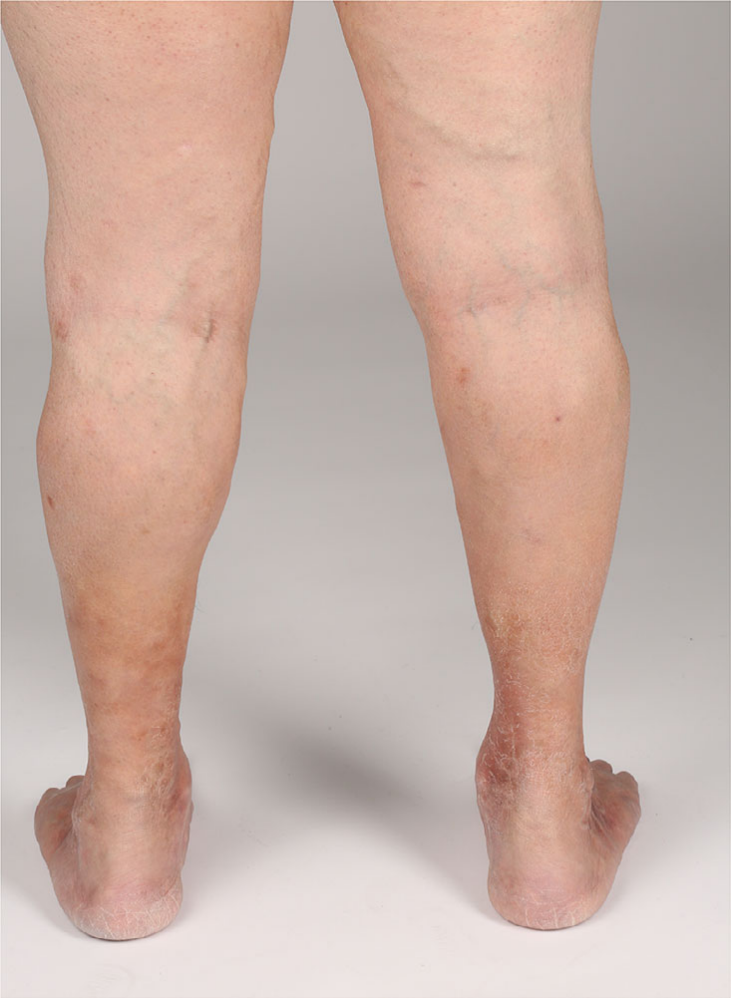
Postoperativer Befund unter noch laufender Sklerosierungstherapie: Reduktion der Seitenastvarikosis und Hyperpigmentierungen der Ober‐ und Unterschenkel beidseits.

Postoperativ erfolgte die Kompressionsbandagierung des rechten Unterschenkels mit elastischen Mittelzugbinden (Salva‐Last^®^) bis zum Folgetag. Kontrollen des Operationsergebnisses erfolgten an den postoperativen Tagen 1 und 8, hier zeigte sich duplexsonographisch ein vollständiger Verschluss der behandelten V. saphena parva. Die Seitenastvarikosis wurde mittels aufgeschäumten Sklerosierungsmitteln (Polidocanol 0,5%, je 1 mL Polidocanol; 4 mL Luft) mit 2 mL pro behandeltem Gefäß durchgeführt und erfolgte erstmalig 2 Wochen nach Operation, mit Beginn an den Oberschenkeln und Fortführung nach distal. Das Aufschäumen erfolgte über einen Dreiwegehahn. Bislang wurden sieben Behandlungstermine komplikationslos durchgeführt, die jeweils einen adäquaten Verschluss der sklerosierten Gefäße zeigten. Vereinzelt traten schmerzhafte Sklerothromben auf, die durch Punktion, Thrombusexpression sowie Heparinsalbenverbände und Fortführung der Kompressionstherapie mit einem medizinischen Kompressionsstrumpf der Klasse II symptomatisch erfolgreich behandelt wurden. Bei der duplexsonographischen Nachuntersuchung 1 Jahr postoperativ zeigte sich die behandelte V. saphena parva weiterhin vollständig verschlossen (Abbildungen 1, 2).

## PATIENTENFALL 2

Im Sommer 2023 stellte sich in unserer ambulanten Sprechstunde eine 64‐jährigen Patientin mit einem vordiagnostizierten cEDS vor. Zu diesem Zeitpunkt bestand eine langjährig bekannte chronische venöse Insuffizienz am linken Bein mit Stammveneninsuffizienz der V. saphena magna sowie Zustand nach multiplen oberflächlichen Beinvenenthrombosen sowohl im Bereich der insuffizienten Stammvene als auch im Bereich angrenzender Seitenäste. Eine operative Therapie war bis zu diesem Zeitpunkt nicht erfolgt. Klinisch präsentierte die Patientin Seitenastvarizen und eine *Purpura jaune d'ocre* am linken Bein. Duplexsonographisch zeigte sich eine komplette Stammveneninsuffizienz der V. saphena magna mit Insuffizienz der saphenofemoralen Terminalklappe (Stadium II–III nach Hach, max. Durchmesser 3 cm und 15 cm distal der saphenofemoralen Crosse: 10,9 mm und 9,5 mm; Stadium C4a).

**ABBILDUNG 3:**
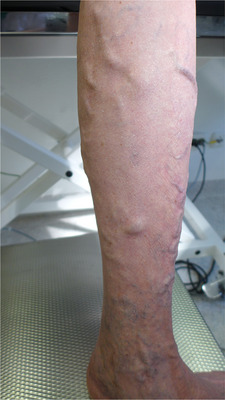
Prätherapeutischer Befund: Großlumige Seitenastvarikosis des linken Unterschenkels mit distal gelegener retikulärer Varikosis und Purpura jaune d'ocre.

**ABBILDUNG 4:**
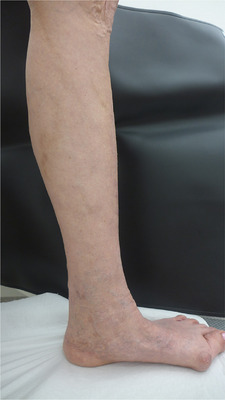
Postoperativer Befund: Deutliche Reduktion der ehemals großlumigen Seitenastvarikosis, der retikulären Varikosis und der Hyperpigmentierungen des linken Unterschenkels.

Aufgrund der Progredienz der chronischen venösen Insuffizienz mit akuten (Oberflächenthrombosen) und chronischen (Hyperpigmentierungen) Komplikationen besprachen wir mit der Patientin die medizinische Indikation einer Sanierung. Vor dem Hintergrund des bekannten EDS entschieden wir uns für eine endoluminale Laserablation der linksseitigen V. saphena magna unter Verzicht auf eine begleitende Miniphlebektomie der Seitenastvarikosis. Der Eingriff erfolgte in Tumeszenzanästhesie mit einem 1940‐nm‐Radiallaser unter Applikation einer Gesamtenergie von 3834,3 J und langsamem Rückzug der Glasfaser über eine Gesamtlänge von 37 cm innerhalb von 454,6 s. Es wurde eine lineare Energiedosis von 160 J/cm für die proximalen 5 cm der Vena saphena magna angestrebt, für die weiter distal gelegenen Abschnitte 80 J/cm. Es zeigten sich keine intraoperativen Komplikationen im Sinne von Gefäßverletzungen oder Blutungen. Postoperativ wurden der linke Ober‐ und Unterschenkel bis zum Folgetag komprimiert und klinische und duplexsonographische Verlaufskontrollen des Operationsgebietes an den Tagen 1 und 8 durchgeführt. Die endoluminal behandelte Stammvene zeigte den gewünschten Verschluss; ausgedehnte Hämatome oder postoperative Schmerzen traten nicht auf. Es erfolgten zwei ambulante Sitzungen zur Sklerosierungstherapie der Seitenastvarikosis mit über einen Dreiwegehahn aufgeschäumtem Polidocanol (0,5%; je 1 mL Polidocanol, 4 mL Luft). Pro Gefäß wurden 2 mL Sklerosierungsmittel injiziert, was bei der Patientin teilweise zu überschießenden Sklerosierungsreaktionen führte. Therapeutisch wurde die Kompressionstherapie fortgeführt und mit regelmäßigen Heparinsalbenverbänden und Eigenmassagen ergänzt, worunter sich die Reaktionen rückläufig zeigten. Bei der letzten Untersuchung 1 Jahr nach der Laserablation zeigte sich ein vollständiger Verschluss der V. saphena magna ohne Reststumpf an der saphenofemoralen Junktion (Abbildungen 3, 4).

## DISKUSSION

Die operative Therapie der chronischen venösen Insuffizienz stellt neben den konservativen Therapieverfahren eine tragende Säule in der Therapie der symptomatischen Varikose dar, insbesondere bei progredienten Beschwerden seitens der Patienten oder komplikativen Verläufen wie (oberflächlichen) Beinvenenthrombosen oder chronischen venösen Ulzera.^6^

Patienten mit EDS sind ein besonderes Kollektiv, da sie aufgrund von Defekten der Bindegewebssynthese bereits in jungen Jahren vermehrt zur Varizenbildung neigen^3^, ^5^ und in der Therapie, insbesondere bei operativen Behandlungen, Risiken für komplikative Verläufe aufweisen. In der Literatur finden sich Einzelfallberichte von Komplikationen bei operativen oder interventionellen medizinischen Eingriffen am arteriellen Gefäßsystem,^2^, ^7^ darunter ein deutlich erhöhtes Risiko der Gefäßverletzungen, wie Gefäßeinrisse, die Bildung von echten oder falschen Aneurysmen, arteriovenöse Fistelbildungen oder ausgeprägte, schwer stillbare Blutungen bis hin zu Extremitätenverlusten oder letalen Ausgängen von Eingriffen.^3^, ^7^, ^8^

Auch bei venösen Eingriffen zeigte sich in Fallberichten eine erhöhte Verletzbarkeit der Gefäße, teils mit Rupturen bei nur geringer Manipulation.^5^, ^8^, ^9^ Ob Patienten mit EDS bei varizenchirurgischen Eingriffen häufiger von transfusionspflichtigen Blutungen betroffen sind, ist derzeit nicht bekannt. Vor diesem Hintergrund scheint es begründet, dass insbesondere beim vEDS offen‐operative Eingriffe vermieden werden,^3^ oder wie in vergangenen Arbeiten postuliert,^5^, ^10^ nur durchgeführt werden, wenn medizinisch unvermeidlich. Interessanterweise gibt es keine aktuelleren Hinweise zum offen chirurgischen Vorgehen in der Bahndlung von Insuffizienzen der Saphenavenen bei Patienten mit EDS. Ältere und allgemeine Empfehlungen sowie Vorsichtsmaßnahmen bei offen‐chirurgischen Eingriffen^7^ umfassen die Ligatur der Saphenavenen in ausreichendem Abstand zum saphenofemoralen Übergang, ein besonders vorsichtiges Vorgehen bei Dissektionen im Leistenbereich aufgrund der Brüchigkeit von Gefäßen und Faszien, den Einsatz von Metallclips bei erschwerten Nahtverhältnissen sowie das längere Belassen von Hautfäden.^7^ Bislang liegen in der Literatur nur zwei Fallberichte zur endoluminalen Varizenbehandlung bei Patienten mit vEDS vor.^3^, ^9^ Ein Fallbericht beschreibt einen 18‐jährigen Patienten mit chronischer venöser Insuffizienz, der erfolgreich mittels Radiofrequenzkatheterablation (ClosureFast™‐Katheter, Covidien) beider Vv. saphenae magnae sowie der V. saphena parva links und durch eine additive Sklerosierungstherapie von Seitenastvarizen behandelt wurde.^3^ Ein weiterer Artikel berichtet über die erfolgreiche Behandlung einer 61‐jährigen Patientin mittels einer Kombination aus endoluminaler Laserablation (ELVeS Radial, Biolitec; LEED 60 J/cm; Radialfaser) und transluminaler Perforansvenenokklusion (TransLuminal Occlusion of Perforator – TRLOP).^9^

Bei zwei unserer Patienten wurde erstmals die endoluminale Lasercrossektomie mittels Radialfaser (1940 nm) an einer insuffizienten V. saphena magna und insuffizienten V. saphena parva in Kombination mit anschließenden Sklerosierungstherapien von Seitenastvarizen erfolgreich eingesetzt. Die bei der Sklerosierungstherapie in beiden Fällen aufgetretenen überschießenden Reaktionen mit vorübergehender Schmerzhaftigkeit stellen aus klinischer Erfahrung verhältnismäßig häufige Komplikationen von Verödungstherapien dar und können auch in der Allgemeinbevölkerung regelhaft beobachtet werden. In der Literatur beschriebene Risiken und Komplikationen minimalinvasiv‐endoluminaler Verfahren bei Patienten mit EDS, die in der Entscheidungsfindung berücksichtigt werden müssen, sind derzeit nur selten dokumentiert. Bei der Verwendung von *bare‐tipped laser fibres* kam es zu Perforationen der Stammvenenwände.^9^ Bei Verwendung von kurzwelligen Lasern (810 nm) finden sich zudem zwei Fallberichte, in denen die Ausbildung von arteriovenösen Fisteln beschrieben wird,^11^, ^12^ wenngleich dies bei Patienten ohne EDS auftrat und somit als allgemeine Komplikation endoluminaler Laserablationen mit kurzwelligen Lasersystemen gewertet werden kann. Ein einzelner Fallbericht beschreibt die Ausbildung einer arteriovenösen Fistel nach Verwendung eines langwelligen (1320 nm) Lasersystems, auch hier bei einer Patientin ohne begleitendes EDS.^13^ Die Ausbildung arteriovenöser Fisteln ist insgesamt als seltene Komplikation zu werten.^12^, ^13^ Inwieweit Patienten mit EDS anfälliger für derartige Komplikationen sind, ist derzeit nicht untersucht. Insgesamt zeigt sich bei der Verwendung langwelliger Laser (1320, 1470, 1500, 1920, 1940 nm) und radialstrahlender Fasern ein geringeres Nebenwirkungsspektrum (unter anderem Schmerzen, Ekchymosen, phlebitische Reaktionen, Nervenläsionen) im Vergleich zu kurzwelligen Lasern (810–980 nm), sodass diese gegenüber den kurzwelligen Lasern und *bare fibres* zu bevorzugen sind.^6^

Die verschiedenen endoluminalen Therapieverfahren stellen bei Patienten mit EDS eine Option jenseits der konservativen symptomatischen Therapie dar, wenn diese aufgrund komplizierter Verläufe oder progredienter Beschwerden nicht mehr ausreicht. Dennoch sollte aufgrund der genetischen Prädisposition zu teils schwerwiegenden Komplikationen während Eingriffen am Gefäßsystem stets eine individuelle Risiko‐Nutzen‐Abwägung durchgeführt werden.^2^, ^7^ Dies ist umso relevanter bei Subtypen mit besonderer Affektion der Gefäßwände, wie dem vEDS.

Die hier vorgestellten Fälle und die Literaturdaten weisen auf Vorteile der Laserablation in Kombination mit der Schaumsklerosierung im Hinblick auf die periprozeduale Sicherheit bei Patienten mit EDS hin. Allerdings gibt es über eine Nachbeobachtung von 1 Jahr hinaus keine Daten zum Langzeitverlauf. Inwieweit das Risiko von Rekanalisierungen und duplexsonographischen wie klinischen Rezidiven von Patienten ohne EDS abweicht, ist bislang noch nicht geklärt. Zu diskutieren ist, ob bei Patienten mit EDS mögliche Nachteile in der Effektivität zugunsten der periprozeduralen Sicherheit endovenös‐ablativer Techniken in Kauf genommen werden sollten.

### Fazit für die Praxis

Die ambulante endoluminale Laserablation mit 1940‐nm‐Laser und Radialfaser erwies sich in unserem Zentrum bei zwei Patienten mit Stammvarikosis der V. saphena magna und V. saphena parva bei EDS als intra‐ und postoperativ komplikationslose Behandlungsmodalität mit adäquatem therapeutischem Ergebnis. Inwieweit die Wellenlänge von 1940 nm gegenüber der weiter verbreiteten 1470‐nm‐Wellenlänge Vorteile bietet, ist derzeit nicht abschließend geklärt. Im Gegensatz zur Radiofrequenzablation, bei der ein Sicherheitsabstand von 2 cm zur tiefen Vene erforderlich ist, ermöglicht die Radialfaser eine mündungsnahe Ablation bis unmittelbar an die Crossenregion. Ob diese Technik langfristig auch hinsichtlich der Effektivität Vorteile gegenüber der Radiofrequenzablation bietet, bleibt offen. Zudem ist das Risiko einer Gefäßperforation, wie es bei *bare fibres* aufgrund der punktuellen Hitzeabgabe beschrieben ist, durch die radiäre Abgabe der thermischen Energie reduziert. Langwellige Lasersysteme mit radiärer Energieabgabe zeigen eine niedrigere Rate an unerwünschten intra‐ und postoperativen Komplikationen. Seitenastvarizen ließen sich in unseren Kasuistiken effektiv und mit vergleichsweise geringem Komplikationsrisiko durch Sklerosierungstherapie behandeln. Aus unserer Erfahrung stellt die Kombination aus endoluminaler Laserablation mit 1940‐nm‐Radialfaser und anschließender Schaumsklerosierung ein sicheres Verfahren zur Therapie der chronischen venösen Insuffizienz bei Patienten mit EDS dar.

## DANKSAGUNG

Open access Veröffentlichung ermöglicht und organisiert durch Projekt DEAL.

## INTERESSENKONFLIKT

Keiner.

## References

[1] Malfait F , Francomano C , Byers P , et al. The 2017 international classification of the Ehlers‐Danlos syndromes. Am J Med Genet C Semin Med Genet. 2017;175(1):8‐26. 28306229 10.1002/ajmg.c.31552

[2] Malfait F , Castori M , Francomano CA , et al. The Ehlers‐Danlos syndromes. Nat Rev Dis Primers. 2020;6(1):64. 32732924 10.1038/s41572-020-0194-9

[3] Frank M , Says J , Denarié N , et al. Successful segmental thermal ablation of varicose saphenous veins in a patient with confirmed vascular Ehlers‐Danlos syndrome. Phlebology. 2016;31(3):222‐224. 25926429 10.1177/0268355515585048

[4] D'hondt S , Van Damme T , Malfait F . Vascular phenotypes in nonvascular subtypes of the Ehlers‐Danlos syndrome: a systematic review. Genet Med. 2018;20(6):562‐573. 28981071 10.1038/gim.2017.138 PMC5993673

[5] Wendorff H , Pelisek J , Zimmermann A , et al. Early venous manifestation of Ehlers‐Danlos syndrome Type IV through a novel mutation in COL3A1. Cardiovasc Pathol. 2013;22(6):488‐492. 23688910 10.1016/j.carpath.2013.04.003

[6] Pannier F , Noppeney T , Alm J , et al. S2k guidelines: diagnosis and treatment of varicose veins. Hautarzt. 2022;73(Suppl 1):1‐44. 35438355 10.1007/s00105-022-04977-8 PMC9358954

[7] Hunter GC , Malone JM , Moore WS , et al. Vascular manifestations in patients with Ehlers‐Danlos syndrome. Arch Surg. 1982;117(4):495‐498. 6895992 10.1001/archsurg.1982.01380280075015

[8] Brearley S , Fowler J , Hamer JD . Two vascular complications of the Ehlers‐Danlos syndrome. Eur J Vasc Surg. 1993;7(2):210‐213. 8462713 10.1016/s0950-821x(05)80766-2

[9] Whiteley MS , Holdstock JM . Endovenous surgery for recurrent varicose veins with a one‐year follow up in a patient with Ehlers Danlos syndrome type IV. Phlebology. 2015;30(7):489‐491. 24714385 10.1177/0268355514531412

[10] Brightwell RE , Walker PJ . Lower limb arterio‐venous fistula as a late complication of phlebectomy in a patient with Ehlers‐Danlos type IV. Eur J Vasc Endovasc Surg. 2011;42(5):696‐698. 21782484 10.1016/j.ejvs.2011.06.044

[11] Timperman PE . Arteriovenous fistula after endovenous laser treatment of the short saphenous vein. J Vasc Interv Radiol. 2004;15(6):625‐627. 15178724 10.1097/01.rvi.00000130166.12122.83

[12] Theivacumar NS , Gough MJ . Arterio‐venous fistula following endovenous laser ablation for varicose veins. Eur J Vasc Endovasc Surg. 2009;38(2):234‐236. 19524461 10.1016/j.ejvs.2009.04.021

[13] Rudarakanchana N , Berland TL , Chasin C , et al. Arteriovenous fistula after endovenous ablation for varicose veins. J Vasc Surg. 2012;55(5):1492‐1494. 22119247 10.1016/j.jvs.2011.09.093

